# Chylothorax following transsternal total thymectomy: a case report

**DOI:** 10.1093/jscr/rjac631

**Published:** 2023-01-10

**Authors:** Fumiaki Kato, Masaki Tomita, Kohei Shimmura, Hideyuki Yoshizumi, Toshihiko Sato

**Affiliations:** Division of Thoracic Surgery, National Hospital Organization Miyakonojo Medical Center, Miyakonojo, Miyazaki, Japan; Division of Thoracic Surgery, National Hospital Organization Miyakonojo Medical Center, Miyakonojo, Miyazaki, Japan; Division of Radiology, National Hospital Organization Miyakonojo Medical Center, Miyakonojo, Miyazaki, Japan; Division of Internal Medicine, National Hospital Organization Miyakonojo Medical Center, Miyakonojo, Miyazaki, Japan; Department of General Thoracic, Breast, and Pediatric Surgery, Faculty of Medicine, Fukuoka University, Fukuoka, Japan

**Keywords:** chylothorax, thymectomy, post-operative

## Abstract

Herein, we report a case of chylothorax following total thymectomy. A 46-year-old woman having an anterior mediastinal tumor underwent a thymectomy via median sternotomy. Seven days after surgery, there was no massive pleural effusion. However, on post-operative day 17, a right massive pleural effusion was detected, and it was diagnosed as chylothorax. She was successfully treated with conservative therapy. Chylothorax following thymectomy is a very rare complication.

## INTRODUCTION

The majority of chylothorax after thoracic surgery might occur following esophagectomy, ligation of patent ductus arteriosus, aortic surgery or pulmonary resection with mediastinal lymph node dissection [1–3]. Chylothorax following thymectomy is very rare, and only a limited number of previous reports exist [3–8].

Herein, we report a case of chylothorax as a complication following transsternal total thymectomy.

## CASE REPORT

**Figure 1:**
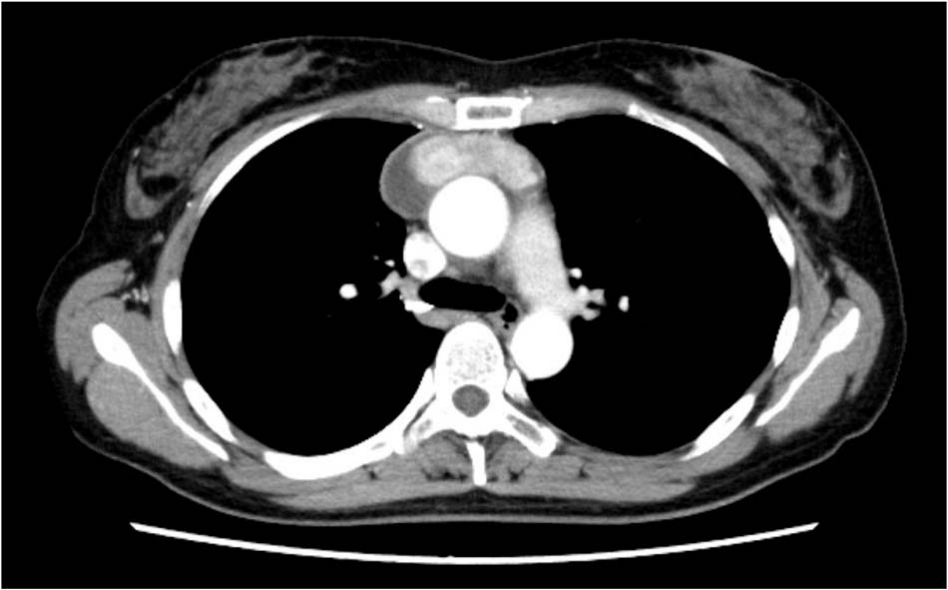
The chest CT scan showing an anterior mediastinal tumor.

A 46-year-old woman presented to a previous hospital with an abnormal shadow on a chest X-ray. Computed tomography (CT) revealed an anterior mediastinal mass lesion with its largest diameter of 6.6 cm (Fig. 1). Therefore, she was referred to our hospital with suspicious mediastinal malignancy. All tumor markers examined were within normal limits.

**Figure 2:**
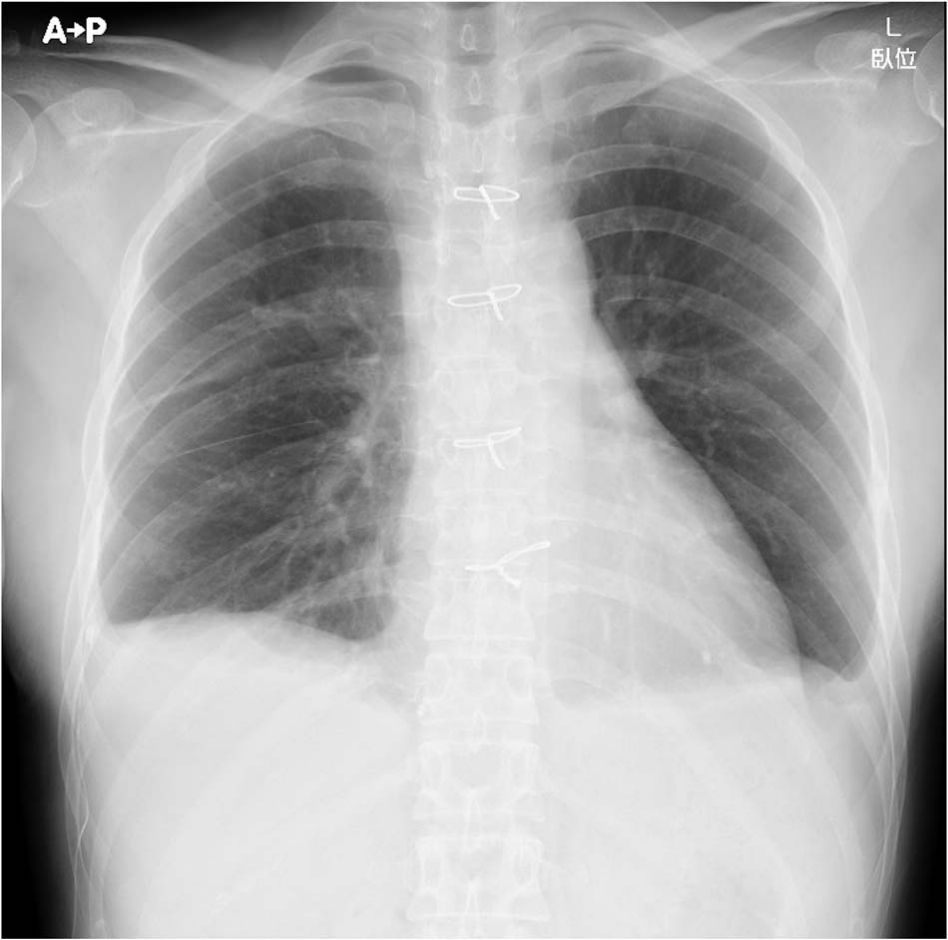
Chest X-ray on post-operative day 7 revealing no massive pleural effusion.

Under general anesthesia, she underwent total thymectomy via median sternotomy. Her post-operative course was uneventful and there was no visible chyle leakage from the chest drainage tube. On post-operative day 2, the chest drainage tube was removed. The chest X-ray on post-operative day 7 revealed no massive pleural effusion (Fig. 2), and she was discharged on the day. The final pathological diagnosis was a Type A thymoma with Masaoka’s Stage I.

**Figure 3:**
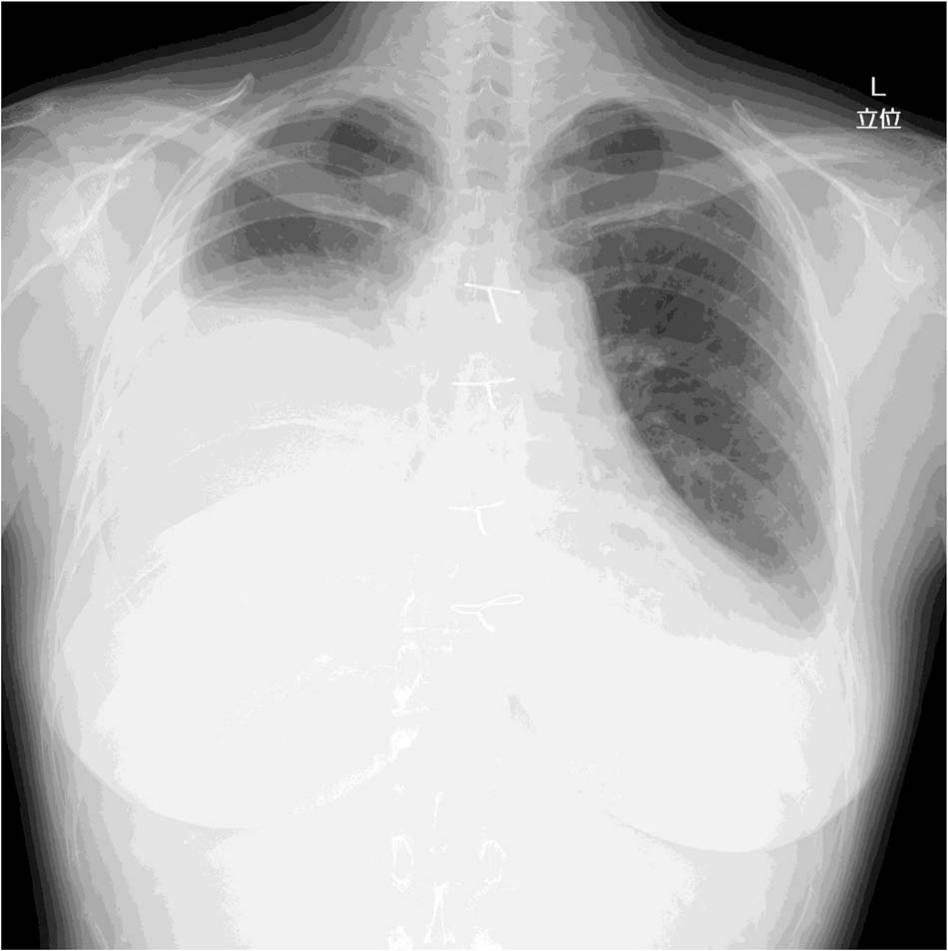
Chest X-ray on post-operative day 17 revealing massive pleural effusion.

On post-operative day 17, she visited our hospital for a routine follow-up, and her chest X-ray revealed a massive right pleural effusion (Fig. 3). Therefore, she was re-admitted to our hospital and right chest drainage was performed. Milky and turbid fluid was drained with an amount of ~3100 ml. The biochemical analysis of pleural fluid revealed a triglyceride level of 1420 mg/dl, and we confirmed a diagnosis of chylothorax. She received fasting and total parenteral nutrition. The fluid via chest tubes returned to be clear with its amount of <200 ml for a day.

**Figure 4:**
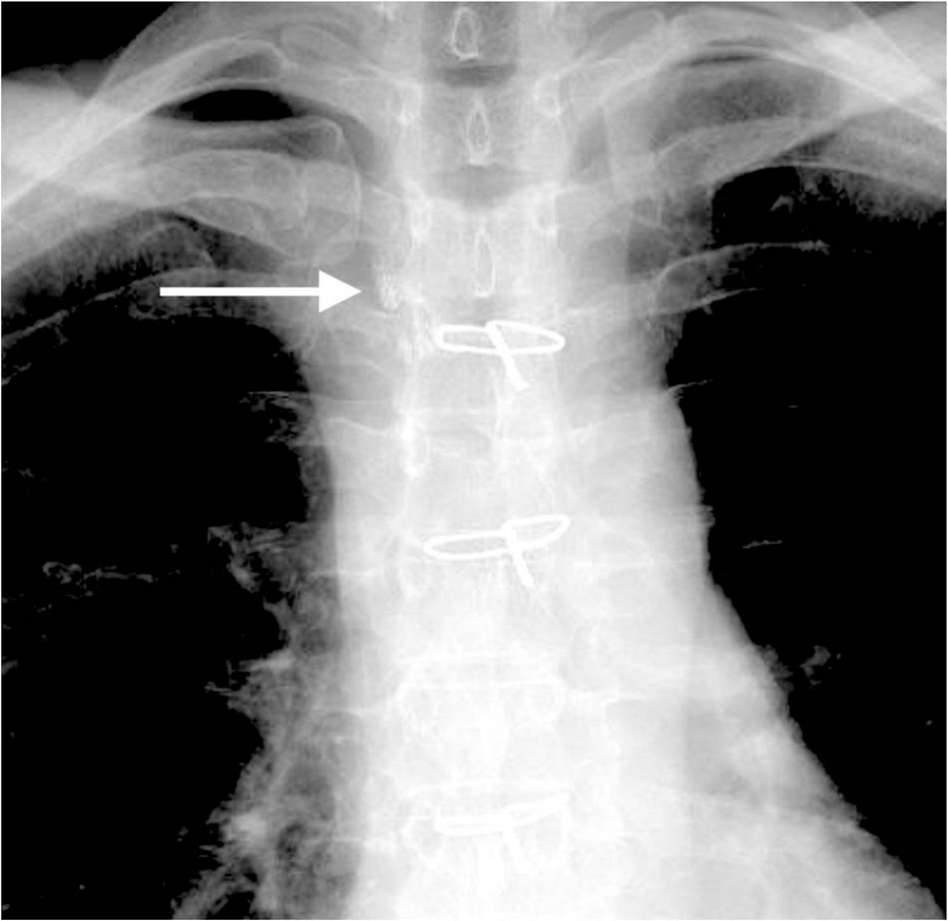
The lipiodol lymphography detected the suspicious location of the chyle leakage (arrow).

Four days after fasting, we resumed the normal diet and pleural fluid changed to slightly milky again. To specifically identify the location of and reduce the chyle leakage, lipiodol lymphography was attempted in the supine position through the right inguinal lymph nodes. The lipiodol lymphography detected the suspicious location of the chyle leakage (Fig. 4). After this procedure, the amount of pleural effusion was further reduced. Chemical pleurodesis with OK-432 was also performed just to be sure. The chest tube was removed the next day. She was discharged 10 days after re-admission. Chylothorax has not recurred 3 months post-operatively.

## DISCUSSION

Chylothorax might occur due to a tear or rupture of the thoracic duct or its branches [1–3]. Among all thoracic procedures, esophagectomy has been reported to be the highest incidence of post-operative chylothorax (1–9%) [2]. However, chylothorax is a rare complication of thymectomy via median sternotomy. It has been reported that the sternal and anterior mediastinal lymphatic drainage system lies anterior and lateral to the thymus as well as just anterior to the great vessels [3, 4]. These small mediastinal lymphatic channels can be accidentally injured intraoperatively, causing post-operative chylothorax [3, 4].

There are only a limited number of previous reports of chylothorax as a complication following thymectomy [3–8]. Zhang *et al.* reviewed 11 cases of chylothorax following thymectomy reported from 1994 to 2017 [5]. They summarized that the majority of patients with chylothorax following thymectomy had myasthenia gravis and transsternal approach and were diagnosed on post-operative 2 or 3 days [5]. Furthermore, these 11 patients were successfully treated with conservative therapy for ~2 weeks, indicating that chylothorax following thymectomy might occur due to an injury of small mediastinal lymphatic channels but not major branches of the thoracic duct [5]. In terms of the present case, she received a transsternal approach, although she did not have myasthenia gravis. The onset of chylothorax in the present case was at least >7 days later, which was later than the 2 or 3 days reported by Zhang [5]. This late onset in the present case is even rarer, and the reason for this is unknown. In the present case, the amount of chest tube drainage was relatively small under fasting and total parenteral nutrition; thus, we considered this complication was caused by the injury of minor lymphatic channels flowing into the thoracic duct. To our knowledge, only three cases of chylothorax following thymectomy [6–8] have been reported since the review by Zhang *et al*. [5]. Among these three patients, two patients were successfully treated with conservative therapy [6, 7], but one received surgery [8].

Conservative management would be the first choice and ideal intervention for post-operative chylothorax patients [1–5]. Recently, conventional lymphangiography has emerged as an important alternative [9]. Lipiodol lymphography enables the detection of the leakage site [9]. In addition to its diagnostic usefulness, the chyle leakage can be occluded by the irrigating effect of lipiodol [9]. We used lipiodol lymphangiography and could obtain an early therapeutic effect. Pleurodesis is an additional non-surgical method for the treatment of chylothorax [2]. The efficacy of using chemical pleurodesis without thoracic duct ligation with success rates has been reported to range from 80 to 100% [2]. Pleurodesis may not have been necessary in the present case, but we added it just in case and got a favorable result.

In conclusion, we reported a rare case of chylothorax following transsternal total thymectomy. Thoracic surgeons should recognize this rare post-operative complication.

## CONFLICT OF INTEREST STATEMENT

None declared.

## FUNDING

None.
